# Verbal memory performance predicts remission and functional outcome in people at clinical high-risk for psychosis

**DOI:** 10.1016/j.scog.2021.100222

**Published:** 2021-10-28

**Authors:** Emily P. Hedges, Hannah Dickson, Stefania Tognin, Gemma Modinos, Mathilde Antoniades, Mark van der Gaag, Lieuwe de Haan, Patrick McGorry, Christos Pantelis, Anita Riecher-Rössler, Rodrigo Bressan, Neus Barrantes-Vidal, Marie-Odile Krebs, Merete Nordentoft, Stephan Ruhrmann, Gabriele Sachs, Bart P. Rutten, Jim van Os, EU-GEI High Risk Study, Lucia R. Valmaggia, Philip McGuire, Matthew J. Kempton

**Affiliations:** a Department of Psychosis Studies, Institute of Psychiatry, Psychology & Neuroscience, King's College London, United Kingdom; b Department of Forensic and Neurodevelopmental Sciences, Institute of Psychiatry, Psychology & Neuroscience, King's College London, United Kingdom; c Department of Psychiatry, Icahn School of Medicine at Mount Sinai, 1425 Madison Ave, New York, NY 10029, United States; d VU University, Faculty of Behavioural and Movement Sciences, Department of Clinical Psychology and EMGO+ Institute for Health and Care Research, van der Boechorststraat 1, 1081, BT, Amsterdam, the Netherlands; e Parnassia Psychiatric Institute, Department of Psychosis Research, Zoutkeetsingel 40, 2512, HN, The Hague, the Netherlands; f Amsterdam UMC, Department Early Psychosis, Meibergdreef 5, 1105, AZ, Amsterdam, the Netherlands; g Arkin Amsterdam, the Netherlands; h Centre for Youth Mental Health, University of Melbourne, 35 Poplar Road (Locked Bag 10), Parkville, Victoria 485 3052, Australia; i Melbourne Neuropsychiatry Centre, University of Melbourne & Melbourne Health, Carlton South, Vic, Australia; j University of Basel, Faculty of Medicine, Basel, Switzerland; k LiNC - Lab Interdisciplinar Neurociências Clínicas, Depto Psiquiatria, Escola Paulista de Medicina, Universidade Federal de São Paulo – UNIFESP, Brazil; l Departament de Psicologia Clínica i de la Salut (Universitat Autònoma de Barcelona), Fundació Sanitària Sant Pere Claver (Spain), Spanish Mental Health Research Network (CIBERSAM), Spain; m University of Paris, GHU Psychiatrie et Neurosciences of Paris, Sainte-Anne, C’JAAD, pôle PEPIT, Inserm 1266, Institut de Psychiatrie (CNRS 3557), Paris, France; n Mental Health Center Copenhagen and Center for Clinical Intervention and Neuropsychiatric Schizophrenia Research, CINS, Mental Health Center Glostrup, Mental Health Services in the Capital Region of Copenhagen, University of Copenhagen, Denmark; o Department of Psychiatry and Psychotherapy, Faculty of Medicine and University Hospital, University of Cologne, Cologne, Germany; p Medical University of Vienna, Department of Psychiatry and Psychotherapy, Austria; q Department of Psychiatry and Neuropsychology, School for Mental Health and Neuroscience, Maastricht University Medical Centre, P.O. Box 616, 6200 MD 464 Maastricht, the Netherlands; r Department of Psychology, Institute of Psychiatry, Psychology & Neuroscience, King's College London, United Kingdom

**Keywords:** Cognition, Verbal fluency, Prodrome, Transition, Early intervention

## Abstract

Robust deficits in cognitive functioning are present in people with psychosis and are evident in the early stages of the disorder. Impairments in verbal memory and verbal fluency are reliably seen in individuals at clinical high-risk for psychosis (CHR) compared to healthy populations. As previous studies have shown a relationship between cognition and longer-term outcomes in schizophrenia, the aim of this paper was to explore whether verbal memory and verbal fluency performance predicted outcomes in a large CHR sample recruited as part of the EU-GEI High Risk Study. Participants included 316 CHR individuals, 90.8% of whom were not currently on antipsychotic medication, and 60 healthy controls. Verbal memory and verbal fluency performance were measured at baseline. At two-year follow-up, CHR individuals were assessed by three different outcome measures, those who did and did not (1) transition to psychosis, (2) experience burdening impairment or disabilities, or (3) remit clinically from CHR status. Individuals with CHR displayed significant verbal memory and verbal fluency deficits at baseline compared to healthy controls (Hedges' *g* effect size = 0.24 to 0.66). There were no significant differences in cognitive performance of those who did and did not transition to psychosis. However, impaired immediate verbal recall predicted both functional disability and non-remission from the CHR state. Results remained significant when analyses were restricted to only include antipsychotic-free CHR participants. These findings may inform the development of early interventions designed to improve cognitive deficits in the early stages of psychosis.

## Highlights

• Cognitive deficits are seen in the early stages of psychosis development.
• Data was collected in the longitudinal, multi-national EU-GEI study.
• Cognition was associated with vulnerability, but not transition, to psychosis.
• Verbal memory predicted non-remission and functional outcome two years later.
• Findings may have implications for early interventions for cognitive impairment.

## 1 Introduction

Cognitive impairment is a core feature of schizophrenia (Sheffield et al., 2018) and is an important predictor of poor functional outcome (Bowie and Harvey, 2005; Green et al., 2004). Deficits in verbal memory and verbal fluency are consistently reported among individuals with schizophrenia (Mesholam-Gately et al., 2009; Schaefer et al., 2013), those experiencing first-episode psychosis (Aas et al., 2014; Mesholam-Gately et al., 2009) and individuals at clinical high-risk for psychosis (CHR) (Bora et al., 2014; Catalan et al., 2021). Although cognitive impairment in CHR is less pronounced than individuals with first-episode psychosis relative to healthy individuals (HC), certain domains, and particularly memory, may be impaired to a comparable degree as first-episode psychosis (Becker et al., 2010; Sheffield et al., 2018). In such instance, verbal memory and verbal fluency performance may be useful in predicting psychosis and functional outcome, as well as targeting early interventions to improve performance (Catalan et al., 2021; Hauser et al., 2017).

Prospective studies of CHR cohorts, where individuals are clinically followed-up after 1–3 years, allow for comparisons of cognitive performance at baseline between CHR individuals who do and do not subsequently (1) transition to psychosis, (2) experience burdening impairment or disabilities, or (3) remit from CHR status. Among individuals who transition to psychosis (CHR-T) compared to those who do not (CHR-NT), existing meta-analytic evidence is inconsistent reporting both significant differences (Bora et al., 2014; Catalan et al., 2021; Fusar-Poli et al., 2012; Hauser et al., 2017) and no differences (Catalan et al., 2021; De Herdt et al., 2013) in verbal memory and verbal fluency performance. In the largest CHR sample of North American Prodrome Longitudinal Study (NAPLS-2), authors reported differences in immediate verbal memory and semantic fluency performance of CHR-T and CHR-NT, but these did not survive Bonferroni-corrections for multiple comparisons (Seidman et al., 2016). Yet, in the latest meta-analysis, impaired verbal memory was the strongest predictor of transition to psychosis (Catalan et al., 2021). In addition, few longitudinal studies have explored individual associations of verbal memory and verbal fluency with CHR functional and remission outcomes. Among them, there have been conflicting findings for verbal fluency and verbal memory for predicting social and occupational functioning (Bolt et al., 2019; Lin et al., 2011; Niendam et al., 2007) and remission outcomes (Addington et al., 2019b; Glenthøj et al., 2021; Lee et al., 2014; Simon et al., 2012). Here, the lack of consensus is likely attributable to limited sample sizes, attrition during follow-up and phenotypic heterogeneity. Although underexplored, it is important to determine cognitive predictors of functional disability alongside clinical outcomes. Long-term functional difficulties are highly prevalent within CHR individuals, irrespective of transition, and they are not addressed by psychological interventions (van der Gaag et al., 2019) so represent an equally important target for prevention (Carrión et al., 2013).

The aims of the present study were to examine (1) differences in verbal memory and verbal fluency in CHR and HC, as well as (2) in CHR-NT and CHR-T, and (3) the relationship between verbal memory and verbal fluency at baseline and two-year functional and remission outcomes in a large CHR sample. The “EU Network of National Schizophrenia Networks study gene-environment interactions” (EU-GEI) is to-date the largest *multi-national* study of 344 CHR individuals (van Os et al., 2014). As a well-characterised and uniquely globally-representative sample, findings from EU-GEI have the potential to provide a better understanding of the role of cognition and its effect on subsequent outcomes in CHR. Furthermore, EU-GEI consists of a predominantly antipsychotic-free CHR cohort; 90.8% of individuals who completed cognitive testing were not on antipsychotic medication, therefore helping to provide robust findings on neurocognition in CHR individuals.

## 2 Methods

### 2.1 Participants

The EU-GEI High Risk study includes 344 CHR individuals and 67 HC. CHR participants were recruited from 11 early detection centres (London, Amsterdam, The Hague, Basel, Cologne, Melbourne, Vienna, Copenhagen, Paris, Barcelona, and Sao Paolo), having been referred by their local mental health service. HC were recruited at four centres; London including GP lists, national postal address file and Gumtree website; Melbourne, by online advertisement; and Amsterdam and The Hague, by Proefbunny website. Ethical approval for EU-GEI study was obtained locally at each site and participants gave written informed consent.

For inclusion criteria of CHR participants, the Comprehensive Assessment of At-Risk Mental States (CAARMS) (Yung et al., 2005) was used to determine whether individuals met at least one of CHR criteria: Attenuated Psychosis Group, Vulnerability Group or Brief Limited Intermittent Psychotic Symptoms Group. Exclusion criteria for all participants were: (1) past/present diagnosis of psychotic disorder, determined by CAARMS and Structural Clinical Interview for DSM Disorders (First et al., 2002); (2) relevant symptoms explained by neurological disorder or drug/alcohol dependency; (3) contraindications to MRI scanning or unwillingness to provide blood/saliva sample, and (3) IQ estimate < 60. HC participants did not meet CHR criteria. CHR and HC individuals were included in the present study if they had completed at least one measure of verbal memory or verbal fluency at baseline. Typical age of participants was 18–35 years but not restricted to due to variation between sites in the age at which persons are accepted by clinical services.

### 2.2 Procedure

Using a naturalistic, prospective design, the EU-GEI study collected multi-modal data at baseline from July 2010 to August 2015. Participants were invited for follow-up assessments at 12 months and 24 months. For CHR-T participants, further assessments were conducted as soon as possible after transition, one-year and two-years later. Face-to-face assessments were carried out by trained researchers. Researchers had passed the online training course which involved rating CAARMS and Global Assessment of Functioning (GAF) training videos. Inter-rater reliability across the EU-GEI centres was assessed from the training videos and scores greater than 0.7 were deemed acceptable (eTable 1).

### 2.3 Baseline demographics and cognitive measures

Participants were assessed at each visit on a wide range of social, cognitive, clinical, imaging and blood-based measures (van Os et al., 2014). Instruments were translated into the language local to each site and subsequently translated back for accuracy. The following descriptions focus only on the measures that are relevant to this paper.

Data on demographic characteristics of participants were collected at baseline (e.g., age, gender) using the Medical Research Council socio-demographic schedule (Mallett, 1997). Socio-economic status (SES) was defined by father's social class at participant's birth. SES was categorised into a three-class model: salariat, intermediate and working class. Fathers who were long-term unemployed were reclassified according to their last main paid job and the never worked/full-time students were excluded (n = 2) (Harrison and Rose, 2006). Intelligence (IQ) was measured using a short version of the Wechsler Adult Intelligence Scale-III, including the four subtests of Block Design, Arithmetic, Digit Symbol, and Information (Blyler et al., 2000), which have been shortened, and demonstrates good reliability and predictive validity (Velthorst et al., 2013).

This paper focuses on two widely-used instruments as measures of verbal memory and verbal fluency. Verbal memory was assessed with the Rey Auditory Verbal Learning Test (RAVLT) (Delaney et al., 1992), which uses a fixed-order 15-word list to measure immediate recall (total number of words correctly recalled for Trials I-V) and delayed recall (total words correctly recalled for Trial VI). Verbal fluency was evaluated using the Verbal Fluency Test (Henry and Crawford, 2005) during which participants are asked to generate as many words as possible in 60 s for a given letter or category. Semantic fluency was defined by the total number of words produced in the animal names subtest; and phonemic fluency (or letter fluency), by the total words produced in the letter subtest. Letter subtests differed between sites to match first letter frequencies within each language where possible (e.g., FAS was utilised for London/Cologne and SNA for Amsterdam/The Hague). Raw scores were converted into standardised *z*-scores based on the HC group (CHR mean minus HC mean, divided by HC standard deviation), so that the HC group had a mean of 0 and standard deviation of 1.

### 2.4 Clinical measures at follow-up

CHR participants were first categorised into CHR-Ts and CHR-NTs. Transition to psychosis was defined as the development of full threshold psychotic disorder using the CAARMS (Yung et al., 2005). Available clinical records were used to determine any diagnosis of a psychotic disorder when participants did not return for follow-up assessments. CHR participants were further grouped into remitters (CHR-R) and non-remitters (CHR-NR). CHR-R participants were those whose symptoms remitted at two-year follow-up (i.e., no longer met CHR criteria). Participants who still met CHR criteria at follow-up or had transitioned to psychosis were categorised as CHR-NRs. Global functioning was also assessed using GAF (Hall, 1995) which was split into disability and symptoms subscales. GAF disability subscale resembles the established Social and Occupational Functioning Assessment Scale (Goldman et al., 1992). GAF scores range from 0 to 100; higher scores indicate superior functioning or fewer symptoms within the last month. Due to natural variation in follow-up visits, analyses for the present paper were restricted to include two-year data that was collected between 1.5 and 2.5 years after baseline.

### 2.5 Statistical analyses

Statistical analyses were performed using IBM SPSS Statistics 26 for Windows. CHR individuals and HC were compared on baseline demographic characteristics using independent *t*-tests for continuous dependent variables and chi-square tests for categorical dependent variables. Baseline characteristics were also compared between CHR who had clinical data at follow-up and CHR who did not complete clinical follow-up measures.

For case-control comparisons, univariate general linear models were implemented to determine any differences in verbal memory and verbal fluency performance. The same model was then utilised to compare baseline cognitive performance in CHR-T and CHR-NT.

In relation to clinical outcomes, general linear model univariate analyses were conducted to determine the effect of baseline cognitive performance in CHR on GAF disability impairment at two-year follow-up. Binary logistic regression was carried out to examine whether baseline cognitive performance in CHR predicts remission at follow-up.

Analyses were repeated for each parameter of verbal memory (immediate and delayed recall) and verbal fluency (phonemic and semantic fluency). These were chosen as the most commonly reported in primary studies for measures of verbal memory and verbal fluency (Fusar-Poli et al., 2012). Statistical significance was set at *p* < 0.05 (two-tailed). In Model 1, analyses accounted for age, site, and gender (Menghini-Müller et al., 2020). For any significant finding, analyses were repeated to include only CHR participants who were not on antipsychotic medication at baseline cognitive assessment (Model 2). SES was included as an additional covariate in Model 3 as this measure differed between groups. It was not included in Model 1 because 14.8% of SES data was missing and may have reduced statistical power; conversely there was no missing data for the other covariates. Confounds were selected *apriori* based on previous research to account for additional variance in the model and increase sensitivity of the analyses. IQ was not added as a covariate. As low IQ has been reported as a risk factor for later development of schizophrenia (Dickson et al., 2012) and IQ is correlated with verbal memory fluency tests, including IQ in statistical analyses may result in an underestimation of impairments in these neurocognitive domains (MacCabe et al., 2012).

## 3 Results

### 3.1 Sample characteristics

The final sample consisted of 316 CHR participants and 60 HC. Table 1 provides information on baseline characteristics of participants. There were significant differences in IQ, years in education, SES and GAF scores of CHR and HC. Most CHR participants (90.8%) were not on antipsychotic medication at the baseline cognitive assessment. A total of 156 CHR participants returned at two-year follow-up. CHR participants who did and did not complete follow-up measures differed significantly at baseline in age and years in education but not in any cognitive or other demographic measure (eTable 2).

**Table 1:** Characteristics of CHR participants and healthy controls at baseline.

	HC (N = 60)	CHR (N = 316)	HC vs CHR (*p*-value)
Age in years, M(SD)	23.68 (4.15)	23.05 (4.96)	0.357
Gender female, n(%)	28 (46.67)	148 (46.84)	0.981
Years in education^a^, M(SD)	16.25 (2.77)	14.42 (3.11)	<0.001⁎⁎
IQ^b^, M(SD)	112.08 (18.01)	98.37 (16.98)	<0.001⁎⁎
SES^c^, n(%)			0.005⁎⁎
Salariat	27 (54.00)	95 (34.17)	
Intermediate	18 (36.00)	101 (36.33)	
Working class	5 (10.00)	82 (29.50)	
GAF symptoms^d^, M(SD)	86.80 (11.03)	55.06 (10.20)	<0.001⁎⁎
GAF symptoms at follow-up^e^, M(SD)	–	62.49 (12.69)	
GAF disability^f^, M(SD)	85.37 (9.11)	55.76 (12.46)	<0.001⁎⁎
GAF disability at follow-up^g^, M(SD)	–	63.16 (14.69)	
Antipsychotic use^h^, n(%)	–	28 (9.20)	
Current cannabis use^i^, n(%)	17 (43.59)	82 (34.89)	0.295

Data was missing for: ^a^24 CHR; ^b^1 HC and 18 CHR; ^c^10 HC and 38 CHR; ^d^1 HC and 16 CHR; ^e^ 203 CHR; ^f^1 HC and 8 CHR; ^g^194 CHR; ^h^10 CHR; ^i^21 HC and 81 CHR.

⁎⁎ *p*-value <0.01.

### 3.2 Baseline cognition group comparisons

**Fig. 1:**
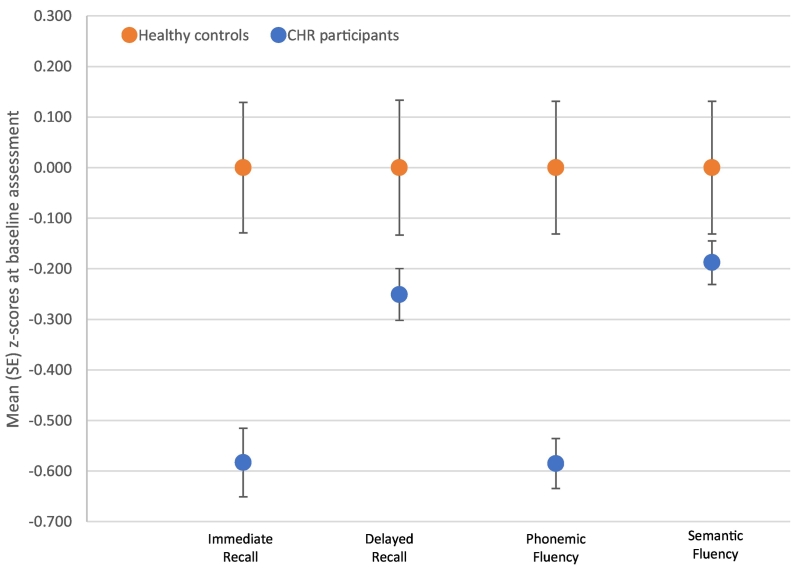
Mean (SE) standardised scores of cognitive performance for CHR participants and healthy controls at baseline.

At baseline, the CHR group performed significantly worse than HC on tests of immediate recall [F(1,359) = 14.43, *p* = 0.00017], delayed recall [F(1,349) = 6.47, *p* = 0.011], phonemic fluency [F(1,367) = 19.12, *p* = 0.00002], and semantic fluency [F(1,366) = 7.34, *p* = 0.007], adjusting for gender, age, and site (Table 2). All findings remained significant in analyses restricted to antipsychotic-free participants and additionally controlling for SES (eTable 3). Fig. 1 displays the mean *z*-scores of performance on each cognitive test for CHR and HC groups.

**Table 2:** Mean (standard deviation) raw scores and between-group comparisons of cognitive performance at baseline.

	HC (N = 60)	CHR (N = 316)	HC vs CHR (*p*-value)	Effect size (Hedges' *g)*	CHR-NT (N = 256)	CHR-T (N = 60)	NT vs T (*p*-value)
*Verbal learning, M(SD)*							
Immediate recall^a^	56.27 (8.50)	51.31 (9.97)	<0.001⁎⁎	0.51	51.26 (9.62)	51.51 (11.49)	0.327
Delayed recall^b^	11.45 (3.48)	10.57 (3.05)	0.011⁎	0.28	10.60 (3.04)	10.45 (3.11)	0.313
*Verbal fluency, M(SD)*							
Phonemic fluency^c^	43.97 (14.43)	35.52 (12.52)	<0.001⁎⁎	0.66	34.73 (12.37)	38.95 (12.67)	0.196
Semantic fluency^d^	22.83 (8.00)	21.32 (6.03)	0.007⁎⁎	0.24	21.33 (5.97)	21.31 (6.37)	0.789

Model 1 for each analysis adjusting for age, gender, and site.

Data was missing for: ^a^12 CHR-NT and 5 CHR-T; ^b^4 HC, 18 CHR-NT and 5 CHR-T; ^c^2 HC, 5 CHR-NT and 2 CHR-T; ^d^2 HC, 6 CHR-NT and 2 CHR-T.

⁎ *p*-value <0.05.

⁎⁎ *p*-value <0.01.

Of 316 CHR participants, 60 participants transitioned to psychosis during follow-up. There were no significant differences seen in immediate recall, delayed recall, phonemic fluency, or semantic fluency scores at baseline between CHR-NT and CHR-T groups (Table 2 and eFig. 1).

### 3.3 Cognitive functioning and clinical outcome

**Fig. 2:**
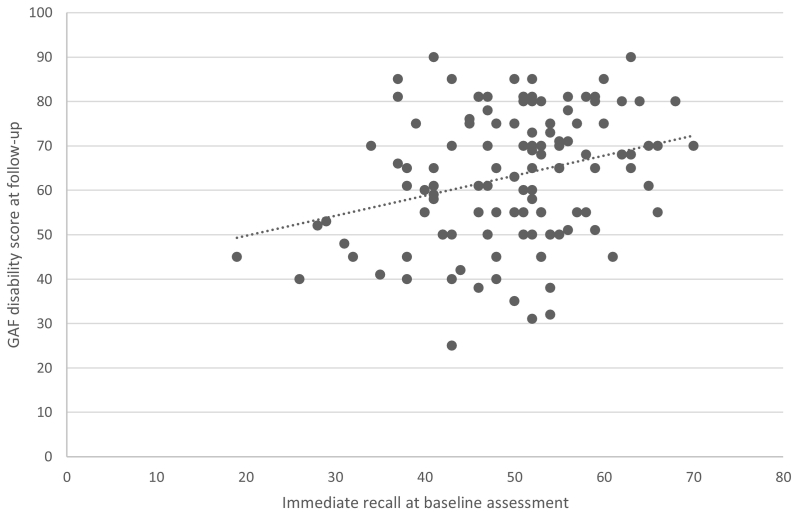
Scatterplot of immediate recall at baseline against GAF disability scores at two-year follow-up in CHR participants.

At follow-up, clinical data was available for 156 CHR participants. Among CHR, immediate recall significantly predicted global disability score [F(1,119) = 5.39, *p* = 0.022] and non-remission [OR = 0.54, 95% CI, 0.34–0.85, *p* = 0.008] at two-year follow-up, controlling for age, gender, and site (Table 3, Table 4, respectively). Fig. 2 shows that higher immediate recall performance at baseline was associated with greater functioning at follow-up. The results did not change in Model 2 or Model 3 (eTable 4).

**Table 3:** Effect of baseline cognition on GAF disability score in 122 CHR at two-year follow-up.

	F	df	Effect size (Cohen's *f*)	*p*-value
*Verbal memory*				
Immediate recall	5.39	1119	0.22	0.022⁎
Delayed recall	0.24	1118	0.05	0.626
*Verbal fluency*				
Phonemic fluency	2.70	1121	0.16	0.104
Semantic fluency	0.62	1121	0.08	0.433

Model 1 for each analysis adjusting for age, gender, and site.

⁎ *p*-value <0.05.

**Table 4:** Effect of baseline cognition on non-remission status at two-year follow-up in CHR participants.

	CHR-R (*N* = 49)	CHR-NR (*N* = 96)	Effect size (odds ratio)	95% CI	*p*-value
*Verbal memory (M, SD)*					
Immediate recall^a^	51.90 (8.62)	49.83 (10.74)	0.54	0.34, 0.85	0.008⁎⁎
Delayed recall^b^	10.42 (2.42)	10.38 (2.95)	0.86	0.46, 1.58	0.621
*Verbal fluency (M, SD)*					
Phonemic fluency^c^	34.16 (12.11)	36.21 (12.86)	0.68	0.40, 1.15	0.152
Semantic fluency^d^	22.43 (6.06)	21.46 (6.34)	0.90	0.50, 1.63	0.722

Model 1 for each analysis adjusting for age, gender, and site.

Data was missing for: ^a^6 CHR-NR and 1 CHR-R; ^b^7 CHR-NR and 1 CHR-R; ^c^2 CHR-NR; ^d^2 CHR-NR.

⁎⁎ *p*-value <0.01.

The findings indicated that delayed recall, phonemic and semantic fluency were not statistically significant predictors of global disability or non-remission at follow-up (Table 3, Table 4).

## 4 Discussion

Our first main finding was that CHR individuals displayed deficits in verbal memory and verbal fluency compared to HC. Our second major finding was that, within the CHR sample, impairments in immediate verbal recall at baseline were associated with both non-remission and functional disability at two-year follow-up. The results remained unchanged following sensitivity analyses with participants who were not on antipsychotic medication.

In line with previous meta-analytic findings, CHR individuals displayed small-to-medium effect size differences in verbal memory and verbal fluency compared to HC (Bora et al., 2014; Catalan et al., 2021; Giuliano et al., 2012). Also, in line with earlier studies, the magnitude of these impairments was smaller than those seen in first-episode psychosis patients relative to controls (standardised mean differences of −1.30 to −0.69) (Mesholam-Gately et al., 2009), indicating that there may be a subsequent decline in performance as the illness progresses. Although such decline is inferred from CHR and first-episode psychosis performance in the same cognitive tasks, the comparison is cross-sectional in nature and so should be interpreted cautiously. Contrary to the results of meta-analyses (Fusar-Poli et al., 2012), we did not observe differences in verbal memory or verbal fluency performance between the CHR-T and CHR-NT groups. Compared to HC, the magnitude of impairment of CHR-T group was similar to that of the overall CHR sample. Therefore, any potential decline in verbal memory and fluency performance was not detectable early on in the prodromal phase of psychosis. Inconsistencies in results between studies may reflect methodological differences (Addington et al., 2019a) such as the use of different memory tasks, which may employ different encoding strategies (Grimes et al., 2017). A further contributory factor may be differences in the nature of CHR samples across centres (Catalan et al., 2021), which vary widely in the way that subjects are ascertained, their average age, and the incidence of transition.

However, poor immediate verbal memory was a predictor of both non-remission from CHR state and greater functional disability at two-year follow-up. To date, few longitudinal studies have explored the association between verbal memory and these two outcomes (Addington et al., 2019b; Bolt et al., 2019; Glenthøj et al., 2021; Lee et al., 2014; Lin et al., 2011; Niendam et al., 2007; Simon et al., 2012) and the findings have been inconsistent. As with transition to psychosis, this lack of consensus may be attributable to the use of different methods for testing verbal memory and defining outcome measures, the use of small sample sizes and variable durations of follow-up. These issues may be addressed by standardising methods across studies and the use of large sample sizes (Addington and Barbato, 2012). Cognition, and particularly verbal memory, is generally viewed as an important contributor of functional impairment in schizophrenia (Green et al., 2000; Kahn and Keefe, 2013) and functional difficulties are highly prevalent in CHR populations (Carrión et al., 2013). Preliminary evidence suggests that cognitive remediation therapies are an effective clinical intervention during clinical high-risk stage (Glenthøj et al., 2017) and are associated with improvements in cognition that enhance functioning in CHR samples (Fiszdon et al., 2016). However, research into other forms of cognitive treatment is also needed (Sheffield et al., 2018). Findings from the present study indicate that verbal memory may be a potentially promising target for early interventions as greater performance in this domain was predictive of remission from CHR state and higher functioning. In addition, cognitive remediation in schizophrenia has the largest effect on improvements in verbal memory as well as in community and work functioning (Lejeune et al., 2021). Future research should examine whether improving verbal memory in CHR reduces the probability of an unfavourable outcome.

The present study has several strengths. First, EU-GEI study collected comprehensive cognitive, clinical, and functional data from a large and globally-represented sample of CHR individuals. Second, the study involves a predominantly antipsychotic-free sample. Therefore, we could explore the role of verbal memory and fluency without the influence of antipsychotic use acting as a confounding variable (Bora and Murray, 2014). Sensitivity analyses confirmed that findings remained significant when the minority (9.2%) of participants on antipsychotic medication were excluded, suggesting that cognitive deficits in CHR may be minimally influenced by current antipsychotic use (Catalan et al., 2021; Pukrop et al., 2006; Seidman et al., 2016). A limitation is that, although typical of similar studies, follow-up lasted for only two years so it is possible that the outcome of CHR individuals may subsequently change (Addington et al., 2019b; Salazar de Pablo et al., 2021). Second, as researchers rated several different clinical scales at follow-up, they were not blinded to transition or remission status. A further limitation is that the study may have been subject to attrition bias. However, baseline cognitive and clinical comparisons of those who did and did not complete follow-up measures reported no differences between the groups.

In conclusion, psychosis is characterised by dysfunctions in several cognitive domains and impairments are reliably reported in verbal memory and verbal fluency (Sheffield et al., 2018). Our findings showed that verbal memory and fluency were associated with vulnerability to psychosis but were not implicated in the subsequent development of the disorder. However, we identified that verbal memory deficits were prominent characteristics of the CHR state and contributed to functional impairment. Therefore, verbal memory may represent an important target for early interventions for cognitive impairment in CHR populations, regardless of who goes on to develop psychosis.

## CRediT authorship contribution statement

**Emily P Hedges:** Conceptualization, Methodology, Software, Validation, Formal analysis, Data curation, Writing – original draft, Writing – review & editing, Visualization.

**Hannah Dickson:** Conceptualization, Methodology, Writing – review & editing.

**Stefania Tognin:** Investigation, Data curation, Writing – review & editing.

**Gemma Modinos:** Investigation, Data curation, Writing – review & editing.

**Mathilde Antoniades:** Investigation, Data curation, Writing – review & editing.

**Mark van der Gaag:** Resources, Data curation, Writing – review & editing, Supervision, Project administration, Funding acquisition.

**Lieuwe de Haan:** Resources, Data curation, Writing – review & editing, Supervision, Project administration, Funding acquisition.

**Patrick McGorry:** Resources, Data curation, Writing – review & editing, Supervision, Project administration, Funding acquisition.

**Christos Pantelis:** Resources, Data curation, Writing – review & editing, Supervision, Project administration, Funding acquisition.

**Anita Richer-Rössler:** Resources, Data curation, Writing – review & editing, Supervision, Project administration, Funding acquisition.

**Rodrigo Bressan:** Resources, Data curation, Writing – review & editing, Supervision, Project administration, Funding acquisition.

**Neus Barrantes-Vidal:** Resources, Data curation, Writing – review & editing, Supervision, Project administration, Funding acquisition.

**Marie-Odile Krebs:** Resources, Data curation, Writing – review & editing, Supervision, Project administration, Funding acquisition.

**Merete Nordentoft:** Resources, Data curation, Writing – review & editing, Supervision, Project administration, Funding acquisition.

**Stephan Ruhrmann:** Resources, Data curation, Writing – review & editing, Supervision, Project administration, Funding acquisition.

**Gabriele Sachs:** Resources, Data curation, Writing – review & editing, Supervision, Project administration, Funding acquisition.

**Bart P Rutten:** Conceptualization, Resources, Data curation, Writing – review & editing, Supervision, Project administration, Funding acquisition.

**Jim van Os:** Conceptualization, Resources, Data curation, Writing – review & editing, Supervision, Project administration, Funding acquisition.

**EU-GEI High Risk Study:** Investigation, Data curation.

**Lucia R Valmaggia:** Conceptualization, Resources, Data curation, Writing – review & editing, Supervision, Project administration, Funding acquisition.

**Philip McGuire:** Conceptualization, Resources, Data curation, Writing – review & editing, Supervision, Project administration, Funding acquisition.

**Matthew J Kempton:** Conceptualization, Methodology, Resources, Data curation, Writing – review & editing, Supervision, Project administration, Funding acquisition.

## Declaration of competing interest

The authors declare no conflict of interest.

## Appendix A Supplementary data

Supplementary Material eTables 1-4 and eFigure 1. Image 1
