# Supplementary material for: Verbal memory performance predicts remission and functional outcome in people at clinical high-risk for psychosis
Source: Schizophr Res Cogn. 2021 Oct 28;28:100222. doi: 10.1016/j.scog.2021.100222 (PMC8861401; doi:10.1016/j.scog.2021.100222)
Supplement: Supplementary file 1 — Supplementary Material eTables 1-4 and eFigure 1. [file mmc1.docx]

# Supplementary Materials

**eTable 1.** Inter-rater reliability analysis of CAARMS and GAF scores

**eTable 2.** Baseline characteristics of CHR individuals who did and did not complete clinical follow-up measures

**eTable 3.** Between-group comparisons of cognitive performance at baseline for Model 2 and Model 3

**eTable 4.** Effect of cognition at baseline in CHR on GAF disability score and remission status at follow-up for Model 2 and Model 3

**eFigure 1.** Mean (SE) standardised scores of cognitive performance for healthy controls, CHR-NT, and CHR-T participants at baseline

**eTable 1:** Inter-rater reliability analysis of CAARMS and GAF scores

| Assessment Scale | Reliability Measurement (Krippendorff’s α) |
| --- | --- |
| GAF | 0.83 |
| CAARMS (Positive items, Intensity scores) | 0.78 |
| CAARMS (Positive items, Frequency scores) | 0.90 |

**eTable 2:** Baseline characteristics of CHR individuals who did and did not complete clinical follow-up measures

|  | CHR with follow-up data (N = 156) | CHR without follow-up data (N = 160) | *p*-value |
| --- | --- | --- | --- |
| Age in years, M(SD) | 23.72 (5.31) | 22.40 (4.51) | 0.018* |
| Gender female, n(%) | 72 (46.2) | 76 (47.50) | 0.811 |
| Years in education^a^, M(SD) | 15.00 (3.00) | 13.84 (3.13) | 0.001** |
| IQ^b^, M(SD) | 99.47 (16.12) | 97.25 (17.80) | 0.261 |
| SES^c^, n(%) |  |  | 0.081 |
| Salariat | 53 (37.06) | 42 (31.11) |  |
| Intermediate | 43 (30.07) | 58 (42.96) |  |
| Working class | 47 (32.87) | 35 (25.93) |  |
| GAF symptoms^d^, M(SD) | 54.19 (8.75) | 55.98 (11.51) | 0.130 |
| GAF disability^e^, M(SD) | 55.35 (10.97) | 56.18 (13.85) | 0.562 |
| Antipsychotic use^f^, n(%) | 11 (7.14) | 17 (11.18) | 0.220 |
| Current cannabis use^g^, n(%) | 43 (35.83) | 39 (33.91) | 0.758 |
| Verbal learning, M(SD) |  |  |  |
| Immediate recall^h^ | 50.60(10.07) | 52.01(9.86) | 0.225 |
| Delayed recall^i^ | 10.46(2.77) | 10.69(3.32) | 0.519 |
| Verbal fluency, M(SD) |  |  |  |
| Phonemic fluency^j^ | 35.78(12.62) | 35.26(12.45) | 0.715 |
| Semantic fluency^k^ | 21.82(6.40) | 20.83(5.62) | 0.150 |

*p < 0.05; ** p < 0.01

Data was missing for: ^a^10 CHR+F and 14 CHR-F; ^b^6 CHR+F and 12 CHR-F; ^c^13 CHR+F and 25 CHR-F; ^d^2 CHR+F and 14 CHR-F; ^e^8 CHR-F; ^f^2 CHR+F and 8 CHR-F; ^g^36 CHR+F and 45 CHR-F; ^h^7 CHR+F and 10 CHR-F; ^i^8 CHR+F and 15 CHR-F; ^j^3 CHR+F; ^k^3 CHR+F and 5 CHR-F

**eTable 3:** Between-group comparisons of cognitive performance at baseline for Model 2 and Model 3

|  | Model 2^a^ | |  | Model 3^b^ | |
| --- | --- | --- | --- | --- | --- |
|  | HC vs CHR (*p*-value) | NT vs T (*p*-value) |  | HC vs CHR (*p*-value) | NT vs T (*p*-value) |
| *Verbal learning* |  |  |  |  |  |
| Immediate recall | <0.001** | - |  | 0.002** | 0.098 |
| Delayed recall | 0.014* | - |  | 0.049* | 0.103 |
| *Verbal fluency* |  |  |  |  |  |
| Phonemic fluency | <0.001** | - |  | <0.001** | 0.595 |
| Semantic fluency | 0.012* | - |  | 0.011* | 0.872 |

*p < 0.05; ** p < 0.01

^a^Model 1 for each analysis excluding all participants who were taking antipsychotic medication at baseline assessment. Model 2 tests were only performed when Model 1 tests were significant to reduce the number of comparisons.

^b^Model 1 for each analysis adjusting for SES

**eTable 4:** Effect of cognition at baseline in CHR on GAF disability score and remission status at follow-up for Model 2 and Model 3

|  | Model 2^a^ | |  | | Model 3^b^ | | |
| --- | --- | --- | --- | --- | --- | --- | --- |
|  | GAF disability (*p*-value) | Remission (*p*-value) |  | GAF disability (*p*-value) | | Remission (*p*-value) | |
| *Verbal learning* |  |  |  | |  | |  |
| Immediate recall | 0.028* | 0.014* |  | | 0.011* | | 0.003** |
| Delayed recall | - | - |  | | 0.377 | | 0.648 |
| *Verbal fluency* |  |  |  | |  | |  |
| Phonemic fluency | - | - |  | | 0.112 | | 0.121 |
| Semantic fluency | - | - |  | | 0.180 | | 0.519 |

*p < 0.05; ** p < 0.01

^a^Model 1 for each analysis excluding all participants who were taking antipsychotic medication at baseline assessment. Model 2 tests were only performed when Model 1 tests were significant to reduce the number of comparisons.

^b^Model 1 for each analysis adjusting for SES

Immediate Recall

Delayed Recall

Phonemic Fluency

Semantic Fluency

**eFigure 1:** Mean (SE) standardised scores of cognitive performance for healthy controls, CHR-NT and CHR-T participants at baseline. Although the graph shows differences in phonemic fluency of CHR-NT and CHR-T groups, the finding is not significant in analyses that adjust for confounding variables.
